# Editorial: Gamification as intervention strategy for neuropsychological rehabilitation

**DOI:** 10.3389/fpsyg.2024.1499643

**Published:** 2024-10-18

**Authors:** Francisco Nieto-Escamez, María Dolores Roldán-Tapia, Sergio Castaño-Castaño, Robert Lowe

**Affiliations:** ^1^Department of Psychology, University of Almeria, Almeria, Spain; ^2^Research Center for Well-Being and Social Inclusion (CIBIS), University of Almeria, Almeria, Spain; ^3^Department of Psychology, University of Oviedo, Oviedo, Spain; ^4^Institute of Neurosciences of the Principality of Asturias (INEUROPA), Oviedo, Spain; ^5^Health Research Institute of the Principality of Asturias (ISPA), Oviedo, Spain; ^6^Department of Applied IT, University of Gothenburg, Gothenburg, Sweden; ^7^RISE Research Institutes of Sweden, Gothenburg, Sweden

**Keywords:** gamification, neuropsychological rehabilitation, cognitive function, virtual reality, therapeutic intervention, exergame, patient engagement

The intersection of technology and health has spurred innovative treatments for neurological conditions, with gamification emerging as a notable strategy in neuropsychological rehabilitation. This concept applies game design elements to therapeutic contexts to boost patient engagement, which is crucial for successful interventions. The immersive nature of games enhances motivation, helping patients take an active role in recovery. Features like goals, feedback, and achievement indicators improve treatment adherence and effectiveness while incorporating social interactions that connect patients with peers and healthcare providers in supportive settings.

Gamification has been adapted for neuropsychological rehabilitation needs, serving individuals with brain injuries and neurodegenerative diseases using tools like mobile apps, virtual reality, exergames, and traditional board games, each tailored to specific therapeutic goals.

This Research Topic focuses on gamification's role in neuropsychological rehabilitation, presenting research that demonstrates its effectiveness and transformative potential. Articles discuss optimizing gamification strategies and emphasize the importance of integrating technological innovations with traditional methods to create engaging rehabilitation practices.

The subsequent sections of this editorial summarize key findings from the five articles included in this Research Topic and discuss the broader implications of gamification in neuropsychological rehabilitation.

In the study by Moret et al., the effects of exergames on mood and cognition in healthy older adults were examined through a randomized pilot study involving 57 participants. Participants were divided into two groups, with one engaging in cognitive-motor exergaming activities on an Xbox360, which included tasks to improve cognitive functions such as visual search, spatial working memory, and executive functions, along with motor skills like proprioception and coordination. Training was conducted over 2–3 weeks, with 3–4 sessions per week, and difficulty levels adjusted based on performance. Post-study analyses indicated a trend toward better scores in information processing speed, working memory, and mood in the exergaming group compared to a passive control group. Participants also reported enjoying the exergames, which may increase adherence to such interventions. The study suggests the need for more robust methodologies to compare exergaming with traditional exercises in enhancing wellbeing and cognitive functions.

Gallagher et al. explored the effects of gamification on impulsivity in children through a gamified Stop-Signal Task (gSST) to test inhibitory control. The study included 30 children, ages 8–12, with four of the children diagnosed with ADHD. They participated in a gSST embedded in a haunted forest game, where they ignored directions given by a witch disguised as a fairy when presented with a stop sign. The study found a moderate correlation between ADHD symptoms and task performance, indicating that ADHD symptoms accounted for 32% of the variance in errors of omission on go trials. However, impulsivity alone did not predict performance, suggesting further investigation is needed with a larger cohort to better understand these relationships.

Gallen et al. investigated how game features added to the Continuous Performance Task (CPT) influence attention and motivation in adults, focusing on participants' ability to maintain focus and respond to stimuli. The study involved 94 participants and explored whether ADHD symptoms, reward responsiveness, and age predict attentional changes when game elements are incorporated into the CPT. The game version of the CPT included competitive elements like a storyline about a fishing competition, allowing participants to compete against avatars and their previous scores. While performance in the game CPT correlated strongly with that in the traditional CPT, indicating a reliance on attentional capacity rather than task features, game elements impaired some performance metrics like processing speed and accuracy. The study found that those with ADHD symptoms, higher reward responsiveness, and younger adults benefited most from the gamified task.

Cano et al. studied cognitive impairments associated with Post-COVID Conditions (PCC) in adults, such as attention deficits and memory loss, and examined the effectiveness of a multimodal Immersive Virtual Reality (IVR) intervention against usual care. The study involved 31 adults from eight primary care centers in Spain, all exhibiting symptoms like brain fog, fatigue, and respiratory problems. The intervention, which lasted 8 weeks with sessions twice per week, combined mindfulness, cognitive exercises, and physical activities in a group setting led by a neuropsychology expert. Although initial sessions showed no significant changes in enjoyment or fatigue, subsequent mixed ANOVA analysis revealed significant improvements in global cognition, processing speed, episodic memory, and depressive symptoms in the experimental group. These findings underscore the potential of multimodal, technology-driven interventions to enhance cognitive and mental health outcomes in PCC patients, offering valuable insights for integrating such methods into healthcare systems during ongoing health crises.

Latella et al. explored the benefits of using Virtual Reality (VR) in rehabilitation for patients with Mild Cognitive Impairment (MCI). The study involved fifty patients from a Neurorehabilitation Unit in Italy, aged around 69.3 years, with an even gender distribution. These participants underwent a neuropsychological evaluation before starting a 5-month intervention program from October 2022 to March 2023, using the VR-based system VESPA 2.0. This system provides immersive 3D VR experiences tailored to improve cognitive functions through activities involving object manipulation, movement, and sensory inputs. Subjects chose their level of immersion based on personal comfort with the technology. The analysis indicated significant improvements in the participants' global cognitive profile, visuospatial skills, and executive functions. However, due to the pilot nature of the study and inconsistent results in previous research, no control group was used. This research underscores the need for validating focused cognitive stimulation interventions for patients with cognitive decline via advanced VR technologies.

Overall, the present studies demonstrate the effectiveness of gamification and immersive technologies in enhancing neuropsychological rehabilitation across diverse conditions and patient groups. Collectively, they show that integrating game elements and VR into therapeutic interventions can improve cognitive functions, patient motivation, and adherence to treatment. These findings highlight the potential of such innovative approaches to not only boost cognitive and motor skills but also enhance mood and mental health. Finally, the studies underline the need for further research to refine these interventions, optimize their implementation, and expand their use in clinical practice, ensuring tailored, engaging, and effective treatment modalities.

